# The Modulation of Interferon Regulatory Factor-1 via Caspase-1-Mediated Alveolar Macrophage Pyroptosis in Ventilator-Induced Lung Injury

**DOI:** 10.1155/2022/1002582

**Published:** 2022-04-15

**Authors:** Minhui Dai, Qian Li, Pinhua Pan

**Affiliations:** ^1^Respiratory Department, Xiangya Hospital, Central South University, China; ^2^Central Hospital, Changsha, Hunan Province, China

## Abstract

**Background:**

To examine the role of interferon regulatory factor-1 (IRF-1) and to explore the potential molecular mechanism in ventilator-induced lung injury.

**Methods:**

Wild-type C57BL/6 mice and IRF-1 gene knockout mice/caspase-1 knockout mice were mechanically ventilated with a high tidal volume to establish a ventilator-related lung injury model. The supernatant of the alveolar lavage solution and the lung tissues of these mice were collected. The degree of lung injury was examined by hematoxylin and eosin staining. The protein and mRNA expression levels of IRF-1, caspase-1 (p10), and interleukin (IL)-1*β* (p17) in lung tissues were measured by western blot and quantitative real-time polymerase chain reaction, respectively. Pyroptosis of alveolar macrophages was detected by flow cytometry and western blotting for active caspase-1 and cleaved GSDMD. An enzyme-linked immunosorbent assay was used to measure the levels of IL-1*β*, IL-18, IL-6, TNF-*α*, and high mobility group box protein 1 (HMGB-1) in alveolar lavage fluid.

**Results:**

IRF-1 expression and caspase-1-dependent pyroptosis in lung tissues of wild-type mice were significantly upregulated after mechanical ventilation with a high tidal volume. The degree of ventilator-related lung injury in IRF-1 gene knockout mice and caspase-1 knockout mice was significantly improved compared to that in wild-type mice, and the levels of GSDMD, IL-1*β*, IL-18, IL-6, and HMGB-1 in alveolar lavage solution were significantly reduced (*P* < 0.05). The expression levels of caspase-1 (p10), cleaved GSDMD, and IL-1*β* (p17) proteins in lung tissues of IRF-1 knockout mice with ventilator-related lung injury were significantly lower than those of wild-type mice, and the level of pyroptosis of macrophages in alveolar lavage solution was significantly reduced.

**Conclusions:**

IRF-1 may aggravate ventilator-induced lung injury by regulating the activation of caspase-1 and the focal death of alveolar macrophages.

## 1. Introduction

Ventilator-induced lung injury (VILI) has been reported in various experimental and clinical settings to potentially cause acute respiratory distress syndrome (ARDS) [1, 2]. Mechanical forces may result in excessive deformation of peripheral lung cells, following inflammatory mediators either directly (released by injured cells) or indirectly (mechanical forces transduced into the initiation of cell signaling pathways), eventually leading to VILI [3–5]. Resident alveolar macrophages (AMs) account for 5% of peripheral lung cells and >90% of leukocytes in bronchoalveolar lavage fluid (BALF) [6] under normal circumstances. Previous literature has described AM activation during mechanical ventilation, accompanied by air-blood barrier dysfunction and VILI. Depletion of AMs in rats attenuated VILI, indicating that AMs may participate in the pathogenesis of VILI [7].

Pyroptosis, a type of programmed cell death, is the process of inflammasome activation and caspase-1/3/4/5/11-dependent cell death [8–11]. In the canonical pathway, following activation and oligomerization of the inflammasome, the caspase-1 zymogen in the inflammasome is cleaved and self-activated. Activated caspase-1 cleaves interleukin (IL)-1 and IL-18 precursors into IL-1*β* and IL-18 and processes gasdermin D (GSDMD) into GSDMD-N and GSDMD-C, resulting in rapid plasma membrane swelling and the release of intracellular proinflammatory contents. With GSDMD-C as inhibitory domain and GSDMD-N as active domain to cause pore-forming and membrane lysis, GSDMD cleaved by caspase-1/4/5/11 at the 272FLTD275 site in pyroptosis [12–14]. Macrophages are major cellular contributors to releasing of proinflammatory cytokines during VILI [15]. Meanwhile, high-mobility group box 1 (HMGB1) translocation, which could induce pyroptosis [16], from macrophages contributes to danger signaling in mediating inflammasome activation and cell death in VILI [17]. However, there is a significant gap in our knowledge concerning the role of AMs pyroptosis in VILI.

Interferon regulatory factor-1 (IRF-1) belongs to a family of highly conserved transcription factors that regulate the expression of specific innate and acquired immune-related genes [18]. IRF-1 has been found to play an essential role in lung injury by modulating the expression of inflammatory mediators [19, 20]. It is also related to the release of inflammatory mediators and pyroptosis of AMs in LPS-related acute lung injury (ALI) [18–20]. The action and the underlying mechanism of IRF-1-mediated pyroptosis in VILI are poorly understood yet.

In this study, we established a VILI mouse model to confirm whether IRF-1 and pyroptosis of AMs were involved in the pathogenesis of VILI. We also explored whether IRF-1 could modulate AM pyroptosis via caspase-1 activation during injurious ventilation.

## 2. Materials and Methods

### 2.1. Animals

Wild-type (C57BL/6J) mice were purchased from SJA Laboratory Animal Co. (Changsha, China), caspase-1 knockout (caspase-1^−/−^) mice were obtained from the Model Animal Research Center of Nanjing University (Nanjing, China), and IRF-1 knockout (IRF-1^−/−^) mice were obtained from The Jackson Laboratory (Bar Harbor, ME, USA). All the mice in this study were male, aged 6–8 weeks, and maintained in the laboratory animal center of the Central South University under specific pathogen-free conditions. The environment has controlled temperature, independent ventilation, and a 12-hour light/dark cycle. All procedures were approved by the Laboratory Animal Ethics Committee of Central South University. All surgeries were performed under a mixture of xylazine and ketamine anesthesia, and all measures were taken to minimize suffering.

### 2.2. VILI Model

The modeling and grouping were performed as described previously, but are briefly explained below. Mice were anesthetized by intraperitoneal (i.p.) injection of ketamine (87.5 mg/kg) and xylazine (12.5 mg/kg) and kept in a prone position on a thermostatic blanket to maintain a temperature of 35 ± 1°C. The anterior neck skin and soft tissue were cut under sterile conditions to expose the trachea to observe the condition of the airway. Orotracheal intubation was then performed with a 20-gauge intravenous catheter (Becton, Dickinson and Company, Piscataway, NJ, USA). The catheter was connected to a ventilator (VentElite; Harvard Apparatus, Holliston, MA, USA) with a fraction of inspired oxygen (FiO_2_) of 0.2 and a volume-controlled setting. Parameters for the low-tidal-volume ventilation and the high-tidal-volume ventilation for 4 h were set as follows: tidal volume of 8 ml/kg body weight with 160 breaths/min and deep inflation with 27 cmH2O for 1 s in every 5 min or 34 ml/kg with 70 breaths/min. Spontaneous efforts were terminated using rocuronium bromide (Esmeron, 0.02 ml/h, i.p., 10 mg/ml) during mechanical ventilation. The sham mice underwent the same surgery and LTV ventilation for 10 min as control mice.

### 2.3. Lung Injury Assessment in Mice

The lung wet-to-dry weight ratio was used as an indicator for the evaluation of pulmonary edema. After the right lower lobe was excised and rinsed quickly in saline, the excess water was drained off the lobe and weighed to determine the wet weight after the mice were killed. The dry weight was determined by weighing the lobe again after drying in an oven at 65°C for 48 h.

The level of protein in BALF was used as an indicator for the evaluation of dysfunction of the alveolar barrier. The protein level in the BALF was evaluated using a BCA protein assay kit (Biomiga, USA) according to the manufacturer's instructions.

For lung histology, a portion of the left lung was fixed with 4% buffered paraformaldehyde and embedded in paraffin, and 6-*μ*m sections were sliced and stained with hematoxylin and eosin. Pathologists blinded to the experimental protocol evaluated and scored the stained sections. The severity of lung injury was scored according to the following indicators: alveolar edema, hemorrhage, alveolar exudates, and leukocyte infiltration.

### 2.4. Isolation of AMs from BALF

After mechanical ventilation/spontaneous breathing, AMs were isolated from the mouse lungs as previously described. In brief, mouse lung was lavaged with 1 mL of sterile saline containing 2% bovine serum albumin and 10 nM ethylenediaminetetraacetic acid disodium through orotracheal intubation, and a total of 10 ml of BALF was collected from each mouse. Leukocytes in the BALF were precipitated by centrifugation at 200×*g* for 10 min at 4°C. AMs in these leukocytes were separated by negative magnetic bead sorting. Magnetic nanoparticle-conjugated antibodies such as antimouse Gr-1, CD4, CD8, and CD45R/B220 antibodies (BD Biosciences Pharmingen, San Diego, CA, USA) were used to label and remove neutrophils and lymphocytes in the immunomagnetic separation system (BD Biosciences Pharmingen). Residual cells were stained and examined by Wright's staining, and the purity of AMs was >95%.

### 2.5. Flow Cytometry

Purified AMs were incubated with Fc block before staining with a fluorescently labeled inhibitor of caspase-1 (FLICA Caspase Assay Kit; ImmunoChemistry Technology, USA) and propidium iodide (ImmunoChemistry Technology) according to the manufacturer's instructions. Flow cytometry analysis was conducted using a FACSVerse BD flow cytometer (BD Biosciences, Sparks, MD, USA). Raw data were analyzed using FlowJo software (TreeStar Corporation, USA). Fluorescently labeled active caspase-1- and propidium iodide-positive cells indicated pyroptosis.

### 2.6. Immunohistochemistry

IRF-1 and cleaved caspase-1 were immunohistochemically stained in paraffin-embedded tissue sections by standard immunohistochemical protocol as described previously [21]. Briefly, pathology slides of lung tissues were incubated with antimouse IRF-1 and caspase-1 P10 (Santa Cruz, CA, USA) at a 1 : 100 dilution. The results were measured by positive cell counts in the field using Leica digital microscopy. All counts were performed by two independent observers to reduce counting bias.

### 2.7. Quantitative Real-Time Polymerase Chain Reaction

The quantification of IRF-1 and caspase-1 was performed as described previously. Trizol reagent (Invitrogen, Carlsbad, CA, USA) was applied to isolate total RNA from AMs. The RNA was converted into reverse transcript (cDNA) using the all-in-one first-stand cDNA synthesis kit (CeneCopoeia, MD, US). The reaction of quantitative real-time PCR (qRT-PCR) was carried out by All-in-One qPCR Mix (CeneCopoeia, MD, US). The reaction system (10 *μ*L) was programmed as follows: 95°C for 10  min followed by 40 cycles at 95°C for 10 s, 60°C for 20 s, and 72°C for 40 s. GAPDH was used as the reference gene. The sequences of primers were as follows: IRF-1 forward: 5′-CTCACCAGGAACCAGAGGAA-3′, reverse: 5′-TGAGTGGTGTAACTGCTGTGG-3′; forward: 5′-ACAAGGCACGGGACCTATG-3′, reverse: 5′-TCCCAGTCAGTCCTGGAAATG-3′; GAPDH forward: 5′-TGCACCACCAACTGCTTAGC-3′, reverse: 5′-GGCATGGACTGTGGTCATGAG-3′.

### 2.8. Protein Extraction and Western Blotting

Cellular and nuclear protein is extracted from AMs as described previously [22]. Total cellular protein extraction was processed with cytoplasmic extraction reagent (Vazyme, China) and protease inhibitor mix. Nuclear protein was isolated by using nuclear extraction reagent (Nanjing, Vazyme, China) with a protease inhibitor mix. Concentration of protein was assessed by a BCA kit (Shanghai, Biyuntian, China). 50 *μ*g protein for western blotting per sample added 4-fold volume of 5*Χ* loading buffer and boiled for 8 min. Protein samples were electrophoresed in sodium dodecyl sulfate-polyacrylamide gels and then transferred to polyvinylidene fluoride (PVDF) membranes (Bio-Rad Laboratories, Berkeley, USA). The PVDF membranes were then incubated with primary antibodies, including antimouse IRF-1 antibody and caspase-1 P10 (Santa Cruz Biotechnology, USA), antimouse GSDMD and histone3 (Abcam, England), GAPDH (ImmunoWay Biotechnology, USA), and HSP90 (Aifang, China) overnight at 4°C after blocking with 5% skimmed milk for 1 h. After three washes with Tris-buffered solution with 0.1% Tween-20, the membranes were incubated with horseradish peroxidase-conjugated secondary antibody (Sigma-Aldrich, USA) for 1 h at room temperature. Signals were detected with a ClarityMax Western ECL Substrate kit (Bio-Rad Laboratories) and were quantified using ImageJ software (Rawak Software Inc., Stuttgart, Germany).

### 2.9. Enzyme-Linked Immunosorbent Assay

The levels of IL-1*β*, IL-6, TNF-*α*, and HMGB-1 in the BALF and serum were measured using commercially available mouse ELISA kits from eBioscience (San Diego, CA, USA). The experimental procedures were performed according to the manufacturer's instructions.

### 2.10. Statistical Analysis

Variables are presented as mean ± standard deviation. Student *t*-test was used for comparisons between the two groups, and one-way analysis of variance was used for more than three groups. Multiple comparisons were corrected using the Bonferroni post hoc test. Correlations between data were assessed using Pearson's correlation analysis. The difference was considered statistically significant when *p* was less than 0.05. All experimental results were repeated at least three times (unless otherwise indicated), and the representative results are shown. The sample sizes (*n*) are indicated in the figures. Statistical analyses were conducted using GraphPad 8 software (GraphPad Software, USA).

## 3. Results

### 3.1. Ventilation with a High Tidal Volume Induces Elevated Caspase-1-Dependent Pyroptosis in AMs

**Figure 1:**
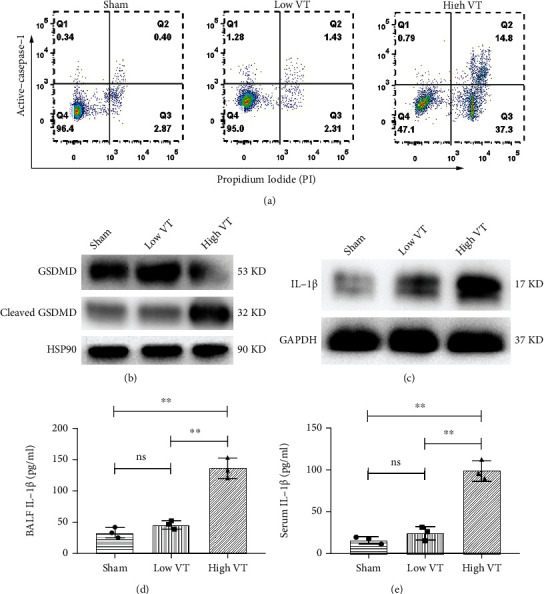
Ventilation with a high tidal volume induces elevated caspase-1-dependent pyroptosis in AMs. Caspase-1-related death cells detected by flow cytometry (a) and the protein level of GSDMD including full-length and cleaved forms (b) and mature IL-1*β* (c) in alveolar macrophages were increased in high VT group compared with the group of control and low VT. The release of IL-1*β* in BALF (d) and serum (e) was significantly increased after ventilation with high VT than that in the low VT group, compared with control group. Results are representative of three independent experiments; the results of one representative experiment are shown (*n* = 5/group, ∗*p* < 0.05, ∗∗*p* < 0.01).

Previously, we demonstrated that lung injury occurred during high-tidal-volume ventilation [23]. To further investigate if AM pyroptosis had occurred, we randomized mice into three groups: a spontaneous breathing control group, a protective ventilation/low-tidal volume ventilation (low VT) group, and an injurious ventilation/high-tidal-volume ventilation (high VT) group. Caspase-1 is a biomarker of canonical pyroptosis. Therefore, we measured the number of active caspase-1-positive and PI-positive to measure caspase-1-related pyroptosis [24]. As illustrated in Figure 1(a), the flow cytometry results shows that percentage of caspase-1-induced pyroptosis was significantly increased in the high VT group, whereas there was no difference between the control group and the low VT group at 4 h after ventilation onset. The same results are verified in western blot as shown in Figure 1(b). The cleaved form of GSDMD, as a biomarker of pyroptosis, increased obviously in the high VT group, but not in low VT groups. The trend of activated caspase-1 was consistent with cleaved GSDMD at the protein level.

In addition, pyroptosis contributes to the mature and release of the proinflammatory cytokine IL-1*β*. The expression of mature IL-1*β* is detected by western blotting, and the release of IL-1*β* is measured by ELISA in BALF and serum and is increased in high VT group compared to that of low VT according to Figures 1(c)–1(e). These results suggest that ventilation with a high tidal volume resulted in elevated caspase-1-dependent pyroptosis in AMs in VILI.

### 3.2. Caspase-1 Deletion Abolishes VILI and Cytokine Release in Mice

**Figure 2:**
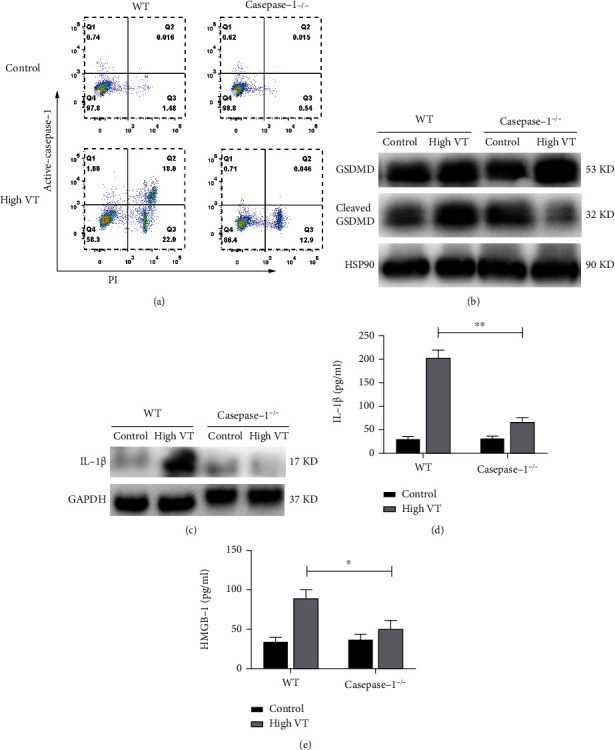
Caspase-1 deletion abolishes VILI and cytokine release in mice. (a) The flow cytometry showed caspase-1 deletion abolished death cells in alveolar macrophage. The protein level of GSDMD including full-length and cleaved forms (b) and mature IL-1*β* (c) in alveolar macrophages was significantly decreased after caspase-1 knockout. Caspase-1 deletion attenuated pyroptosis-related cytokines in BALF of high VT, including IL-1*β* (d) and HMGB-1 (e). Results are representative of three independent experiments; the results of one representative experiment are shown (n =5/group, ∗*p* < 0.05, ∗∗*p* < 0.01).

To investigate whether alveolar pyroptosis contributes to VILI, caspase-1^−/−^ mice were ventilated with a high tidal volume. As shown in Figure 2(a), caspase-1^−/−^ mice that underwent high-tidal-volume ventilation had barely any AM caspase-1-induced pyroptosis and drastic reduction in the proportion of death cells. The expression alteration in GSDMD could be as a supporting information for AM pyroptosis (Figure 2(b)). Protein level of cleaved GSDMD was significantly reduced after caspase-1 knockout. To assess pyroptosis-related inflammatory factor, we found that IL-1*β* and HMGB-1 in BALF was significantly increased in high VT group, but sharp decrease upon caspase-1 knockdown (Figures 2(c)–2(e)).

**Figure 3:**
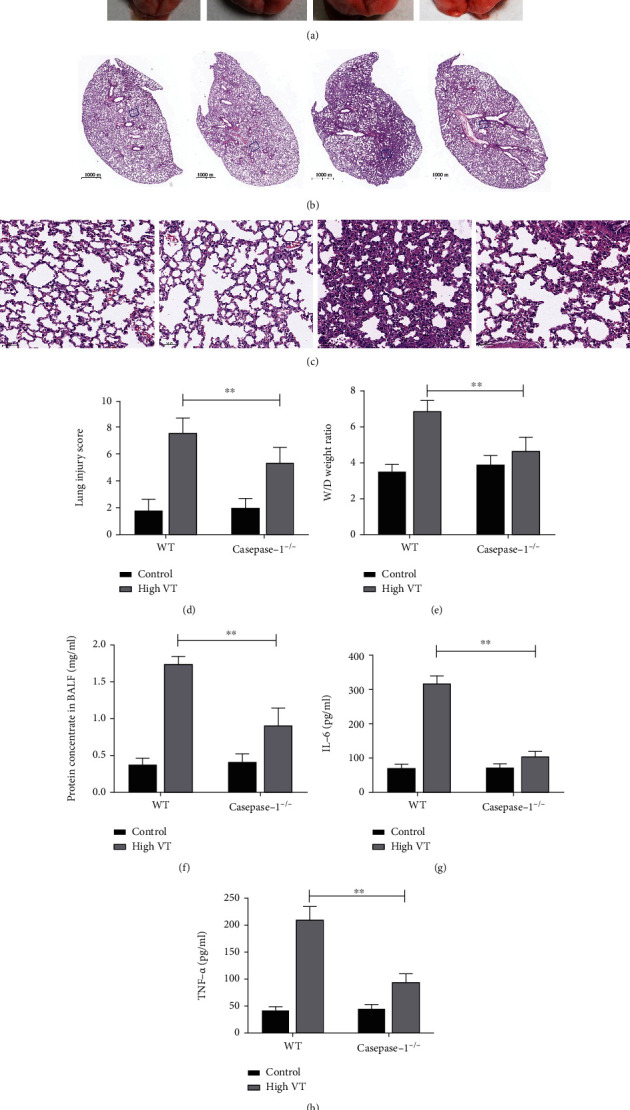
Caspase-1 deletion abolishes VILI and cytokine release in mice. Caspase-1 deletion alleviated the high-tidal-volume ventilation-induced lung injury measured by lung injury scores (d) for lung pathology. (a) Lung gross pathology is shown. Representative histologic sections for lung pathology (b) (magnification, 20*×*) and (c) (magnification, 400×) are shown. Caspase-1 deletion alleviated lung pathology in gross pathology (a) and HE-stained micrographs (b and c). Caspase-1 deletion reduced the wet/dry (W/D) ratio (e) and BALF protein concentration (f) in the high VT group. Caspase-1 deletion attenuated the release of IL-6 (g) and TNF-*α* (h) in BALF of high VT. Results are representative of three independent experiments; the results of one representative experiment are shown (*n* = 5/group, ∗*p* < 0.05, ∗∗*p* < 0.01).

As shown in Figures 3(a)–3(d), the high-tidal-volume ventilation caused significant lung inflammation, alveolar congestion, alveolar septal thickening, and perivascular infiltration of inflammatory cells, whereas lung lesions showed significantly reduced inflammatory cell infiltration in the caspase-1^−/−^ mice. Genetic caspase-1 deficiency significantly alleviated the wet weight/dry weight ratio and reduced the total proteins in the BALF with high VT (Figures 3(e) and 3(f)), which was consistent with our histopathological analysis. To further assess lung injury, we evaluate the levels of IL-6 and TNF-*α* in BALF and shown as Figures 3(g) and 3(h). These cytokines increased dramatically in the wild-type mice that received high-tidal-volume ventilation (high VT group), but cytokines were partially reduced in the caspase-1^−/−^ mice. These findings indicate that genetic caspase-1 deficiency decreases lung damage in VILI in mice.

### 3.3. IRF-1 Deletion Attenuates VILI and Cytokine Release in Mice

**Figure 4:**
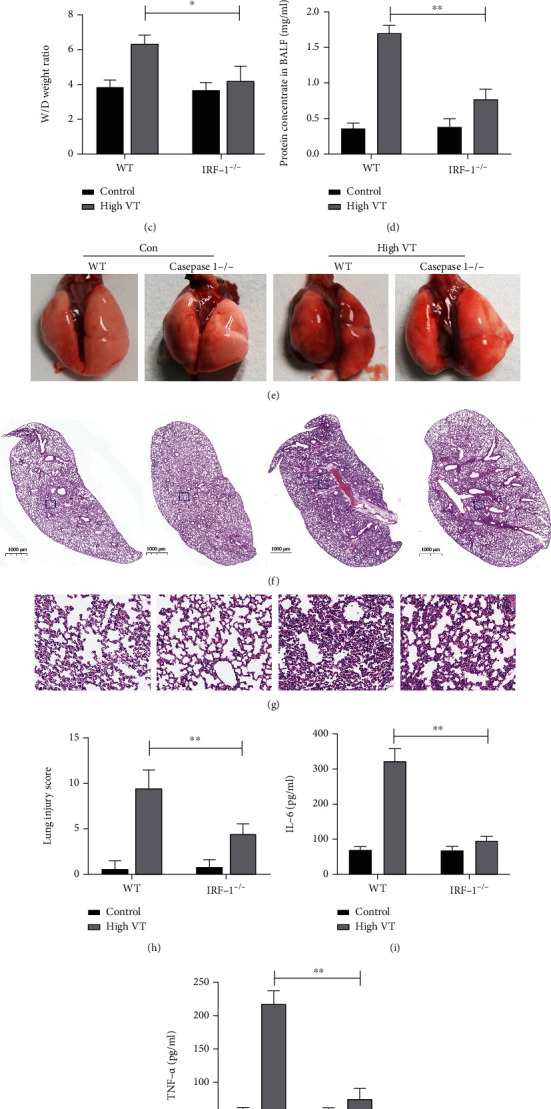
IRF-1 deletion attenuates VILI and cytokine release in mice. The intranuclear protein level (a) and mRNA (b) of IRF-1 in alveolar macrophages were increased in the high VT group compared with the control group and the low VT group. IRF-1 deletion alleviated lung histopathologic damage in gross pathology (e), HE-stained micrographs for 20× magnification (f) and 400× magnification (g) induced by the high-tidal-volume ventilation assessed using lung injury scores (h). IRF-1 deletion alleviated the wet/dry (W/D) ratio (c) and BALF protein concentration (d) in the high VT group. The concentration of IL-6 (i) and TNF-*α* (j) in BALF was attenuated after high VT with IRF-1 deletion compared to that wild type. Results are representative of three independent experiments; the results of one representative experiment are shown (*n* = 5/group, ∗*p* < 0.05, ∗∗*p* < 0.01).

We previously identified that IRF-1 has been implicated in the regulation of ALI-induced inflammatory response [21, 22]. To examine the functions of IRF-1 in VILI, we first examined its RNA and protein content in the group of low VT, high VT, and sham (Figures 4(a) and 4(b)). The expression of IRF-1 in lung homogenates was significantly increased in the high VT group compared with the sham and low VT group.

Next, IRF-1^−/−^ mice were also used to investigate whether IRF-1 mediates VILI and cytokine release. As shown in Figures 4(e)–4(h), lung lesions showed significantly reduced inflammatory cell infiltration in IRF-1^−/−^ mice. Genetic IRF-1 deficiency significantly alleviated the wet weight/dry weight ratio and reduced the total proteins in the BALF (Figures 4(c) and 4(d)–4(f)), which was consistent with our histopathological analysis. To further assess lung injury, we evaluated the levels of IL-6 and TNF-*α* in BALF. All cytokines increased dramatically in the wild-type mice that received high-tidal-volume ventilation (high VT group), but cytokines were partially reduced in the IRF-1^−/−^ mice in Figures 3(i)–3(j). These findings indicate that genetic IRF-1 deficiency decreases lung damage in VILI in mice. These data indicate that IRF-1 plays an important role in the pathogenesis of VILI.

### 3.4. IRF-1 Was Required for Caspase-1 Activation in AMs

**Figure 5:**
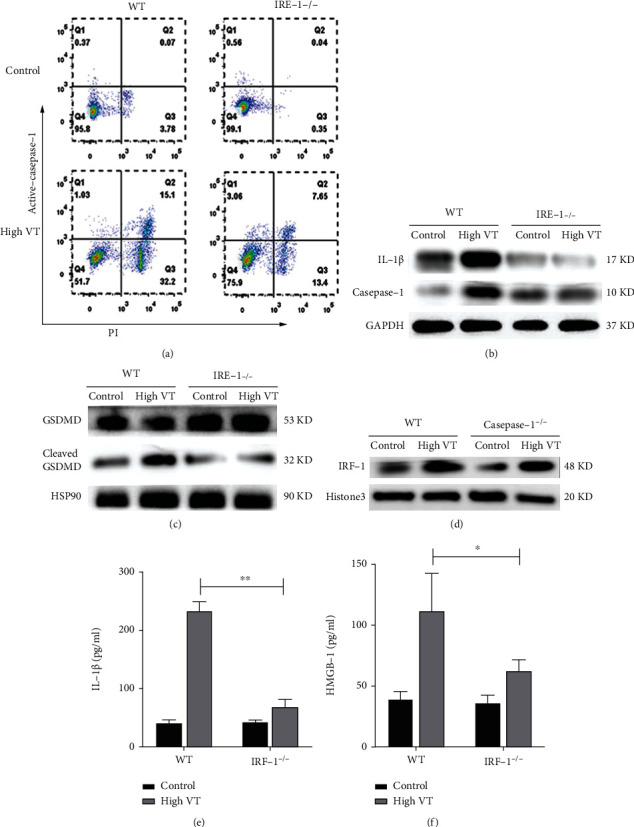
IRF-1 was required for caspase-1 activation in AMs. The flow cytometry showed IRF-1 deletion attenuated alveolar macrophage pyroptosis in high VT (a). Western blotting analysis of protein expression of caspase-1 p10 (b), IL-1*β* (b), and GSDMD including full-length and cleaved forms (c) in alveolar macrophages. Analysis of IRF-1 protein levels (d) in the nucleus of alveolar macrophages indicated that caspase-1 deletion did not affect expression of IRF-1. The concentration of IL-1*β* (e) and HMGB-1 (f) in BALF was attenuated after high VT with IRF-1 deletion compared to that wild type. Results are representative of three independent experiments; the results of one representative experiment are shown (*n* = 5/group, ∗*p* < 0.05, ∗∗*p* < 0.01).

Having shown that VILI is associated with pyroptosis of AMs and IRF-1 expression, we then investigated whether IRF-1 deletion enhances protection by inhibiting AM pyroptosis. As shown in Figure 5(a), IRF-1-/- mice that underwent high-tidal-volume ventilation had very little caspase-1-induced pyroptosis. The levels of activated caspase-1, cleaved GSDMD, and IL-1*β* were detected by western blot analysis. Indeed, reduced expression of cleaved caspase-1, cleaved GSDMD, and IL-1*β* was observed in AMs of IRF-1^−/−^ mice with high-tidal-volume ventilation compared to that of the control group (Figures 5(b) and 5(c)). The levels of IRF-1 are detected by western blot analysis as shown in Figure 5(d). Indeed, reduced expression of IRF-1 was observed in AMs of caspase-1^−/−^ mice with high-tidal-volume ventilation compared to those of the control group. The concentration of IL-1*β* and HMGB-1 in BALF was attenuated after high VT with IRF-1 deletion compared to that wild type (Figures 5(e) and 5(f)). These data indicated that IRF-1 was essential for caspase-1 activation and further precipitated the pathogenesis of VILI.

## 4. Discussion

It has been identified that inhibition or knockout of caspase-1 or IRF-1 has a protect effect against many inflammatory diseases [25–27]. In this study, we demonstrated that caspase-1-related pyroptosis may be an important mechanism in pathogenesis for experimental VILI. Moreover, IRF-1 may positively regulate caspase-1-dependent pyroptosis and release of inflammatory factors in mechanical lung injury. Therefore, our study found accumulating evidence for the links between IRF-1 and pyroptosis-related molecules [28].

Clinically, mechanical ventilation is the most dominant treatment strategy for ARDS. VAP is one of the most common complications in severe pneumonia and ARDS patient. The alveoli damage by mechanical force could further complicate the condition and prognosis of ALI/ARDS. Excluding infection, injurious mechanical ventilation only could induce AM pyroptosis and be associated by caspase-1 in our study. From our findings, caspase-1-dependent pyroptosis potentiates inflammatory response in VILI.

Prior studies have identified the pivotal role of IRF-1 in mechanism of ALI/ARDS occurrence. As a transcription factor that is involved in tumor-related signaling pathways, IRF-1 is often elevated in patients with ARDS [29]. In addition, we found that IRF-1 deletion in LPS-induced ALI mouse could alleviate lung injury significantly [21, 22]. These studies proposed that IRF-1 plays a critical role in mediating cytokine storm of ALI/ARDS. No IRF-1-related signaling pathway contributing to VAP or VILI has been studied before. In our study, IRF-1 was significantly upregulated in AMs in high VT-induced lung injury. Moreover, caspase-1-induced pyroptosis of AMs and inflammation was impaired after IRF-1 knockdown. IRF-1 seems to be upstream in caspase-1 and pyroptosis-related molecules. In other words, it suggested that IRF-1 is a potential transcription factor implicated in caspase-1-related pyroptotic cell death. Previous studies showed that caspase-1 gene might be regulated by IRF-1 via a CRE site. Additionally, caspase-1 upregulation was unable to be observed in oligodendrocyte progenitor cells after IFN stimulation in absence of IRF-1 [30, 31].

In mechanical ventilation-induced lung injury, there were fewer inflammatory factors in serum and BALF. It was distinct from the pathophysiological processes of LPS-induced ALI which could amplify the inflammatory response at the beginning of onset. Nonetheless, caspase-1-induced pyroptosis and the release of related inflammatory factors including IL-1*β*, IL-18, and HMGB-1 were partially responsible for pulmonary pathology in VILI. It has been confirmed that myeloid differentiation factor 88 (MyD88) adapter protein could recruit some members of IRF-1 family of transcription factors to evoke certain genes such as toll-like receptor (TLR) [32, 33]. In our study, IRF-1 knockdown could markedly reduce these effects mentioned above. It appears that IRF-1 regulate pyroptosis-associated cytokines.

In conclusion, our study highlights the important role of caspase-1 and the promoting effect of IRF-1 in the pathogenesis of VILI. IRF-1 and pyroptosis-related inflammatory factors promise to be therapeutic targets or early warning signals in patients undergoing mechanical ventilation. However, our animal experiment may further verify by a prospective clinical study.

## Data Availability

Available upon reasonable request by contacting the corresponding author.
